# Evidence for Variation in the Effective Population Size of Animal Mitochondrial DNA

**DOI:** 10.1371/journal.pone.0004396

**Published:** 2009-02-09

**Authors:** Gwenael Piganeau, Adam Eyre-Walker

**Affiliations:** 1 UPMC Univ Paris 06, UMR 7628, MBCE, Observatoire Océanologique, Banyuls/mer, France; 2 CNRS, UMR 7628, MBCE, Observatoire Océanologique, Banyuls/mer, France; 3 Centre for the Study of Evolution, School of Life Sciences, University of Sussex, Brighton, United Kingdom; University of Otago, New Zealand

## Abstract

**Background:**

It has recently been shown that levels of diversity in mitochondrial DNA are remarkably constant across animals of diverse census population sizes and ecologies, which has led to the suggestion that the effective population of mitochondrial DNA may be relatively constant.

**Results:**

Here we present several lines of evidence that suggest, to the contrary, that the effective population size of mtDNA does vary, and that the variation can be substantial. First, we show that levels of mitochondrial and nuclear diversity are correlated within all groups of animals we surveyed. Second, we show that the effectiveness of selection on non-synonymous mutations, as measured by the ratio of the numbers of non-synonymous and synonymous polymorphisms, is negatively correlated to levels of mitochondrial diversity. Finally, we estimate the effective population size of mitochondrial DNA in selected mammalian groups and show that it varies by at least an order of magnitude.

**Conclusions:**

We conclude that there is variation in the effective population size of mitochondria. Furthermore we suggest that the relative constancy of DNA diversity may be due to a negative correlation between the effective population size and the mutation rate per generation.

## Introduction

Two observations particularly puzzled early workers in the field of molecular evolution. First, why the rate of molecular evolution is relatively constant across species and time, and second, why levels of allozyme diversity vary by no more than a few fold across almost all species [1], [2], [3]. The fact that allozyme diversity varies remarkably little across species is surprising, particularly under the neutral theory of molecular evolution, because under this theory, levels of diversity are expected to be proportional to the effective population size of the organism. Since some organisms differ massively in their census population sizes, one might reasonably expect them to differ considerably in their effective population sizes, and hence to have very different levels of allozyme diversity, and yet they do not. For example, the allozyme diversiy of the mussel *Mytilus edulis* is only about 3-fold greater than the diversity of gorillas (0.095 versus 0.036) [3] and yet there must be millions more mussels in the world than gorillas.

While the molecular clock has been studied in great detail over the last 30 years (reviewed in [4]), the relative constancy of allozyme diversity levels has received almost no attention since the 1970s, (though see [5]). However, Bazin *et al.* [6] have recently published data which has brought this surprising observation back to our attention. Bazin *et al.* [6] showed, for the first time, that levels of sequence diversity, in mitochondrial DNA, are remarkably constant across species that apparently have very different census population sizes; for example they showed that several groups of animals including mammals and molluscs, have very similar levels of mitochondrial DNA sequence diversity. They also showed that there is no apparent difference in the diversity levels of marine and freshwater fish, and marine and terrestrial molluscs - we might expect marine organisms to have much larger population sizes than non-marine organisms. In contrast levels of allozyme diversity and DNA sequence diversity in nuclear DNA do follow the expected pattern, although the differences are modest; for example molluscs have about four-fold more diversity in nuclear loci than mammals [6].

Although there appears to be no relationship between census population size and mtDNA sequence diversity across these very different animal groups, there does appear to be variation in the effective population size of mtDNA, in mammals at least. There are two lines of evidence for this. First, Popadin *et al.* [7] have shown that the ratio of the non-synonymous to synonymous substitution rate, ω, in mtDNA is negatively correlated to body size in mammals; since we expect body size and population size to be negatively correlated, this relationship suggests that ω is negatively correlated to population size which is consistent with there being variation in the effective population size of mtDNA. Second, Mulligan *et al.* [8] and Nabholz *et al.* [9] have shown that there is a correlation between allozyme and mtDNA sequence diversity within mammals; this correlation is consistent with there being correlated variation in the effective population sizes of the nuclear and mitochondrial genomes [9]. Here we extend the analyses of Mulligan *et al.* [8] and Popadin *et al.* [7] to investigate whether there is variation in the effective population size of mitochondrial DNA within other groups of animals. We use two analyses to investigate this question. First, we test whether there is a correlation between levels of allozyme and mtDNA diversity in a diversity of animals. Second, we test whether the level of purifying selection on non-synonymous mutations is correlated to levels of synonymous diversity in mitochondrial DNA. If there is variation in the effective population size of mtDNA then we expect species with low effective population size to show low diversity and relatively inefficient selection against non-synonymous mutations.

## Results

**Figure 1:**
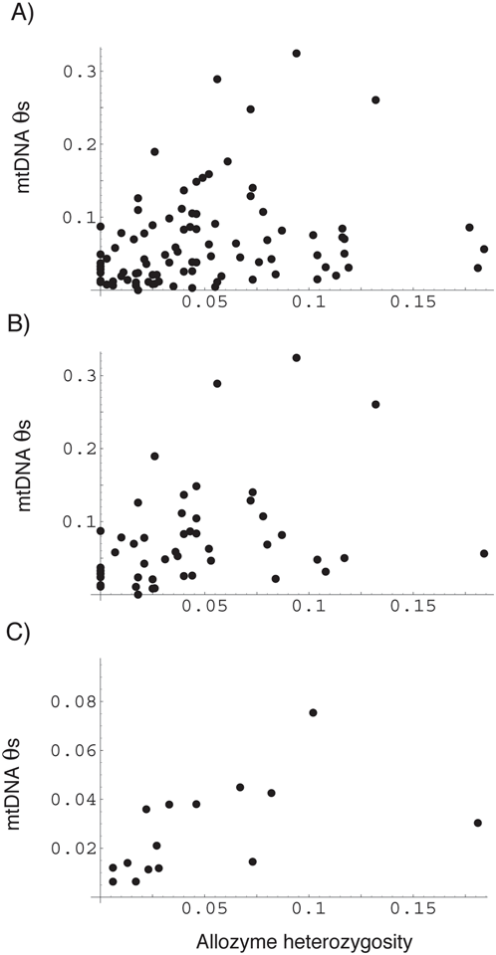
The correlation between synonymous site diversity in mtDNA and allozyme heterozygosity across (a) all species, (b) within mammals, and (c) within fish.

If there is variation in the effective population size of mtDNA across species then we might expect levels of diversity in mtDNA to be correlated to that in nuclear DNA, since many of the processes that affect the effective population size, are likely to affect both the nuclear and mitochondrial genomes. We do indeed observe a correlation between mitochondrial synonymous diversity and allozyme diversity both across the whole dataset and within each group of organisms (Table 1, figure 1), with many of the correlations being significant or nearly significant. Only fish and mammals are significant if we correct for multiple tests, but overall there is a significant correlation for the remaining four groups if we combine probabilities (p = 0.0024). The correlations are also consistently positive in all groups if we control for phylogenetic non-independence by considering pairs of independent taxa. However, we have relatively little data and this correlation is only significant overall and within fish, and the result for fish is not significant if we correct for multiple tests. However, the correlation is positive in all 6 comparisons, which itself is significant (probability of 6 out of 6 correlations being positive by chance is 0.016), and the combined probability across the six datasets is also significant (p = 0.014). However, it is important to appreciate that while there is a significant correlation between levels of mitochondrial and allozyme diversity, the overall variation in both mitochondrial and allozyme diversity is limited; neither diversity varies by much more than one order or magnitude across species.

**Table 1:** The correlation between allozyme heterozygosity and synonymous site diversity in mtDNA across and within several groups of animals.

Dataset	All species			Phylogenetically Independent		
	n	r_s_	p-value	n	r_s_	p-value
All	97	0.36	0.0003	38	0.44	0.006
Amphibians	7	0.18	0.70	3	1.0	0.33
Birds	5	0.90	0.037	2	1.0	1.0
Fish	16	0.71	0.0022	7	0.86	0.014
Insects	6	0.60	0.21	3	0.50	1.0
Mammals	47	0.41	0.0046	19	0.15	0.53
Reptiles	12	0.56	0.056	4	0.80	0.20

The correlation is measured by Spearman's rank correlation coefficient. The number of data-points, *n*, is also given. Note the number of species in each group does not add up to the total number of species since there are some groups not listed, which have one or two species in them.

The positive correlation between mitochondrial and allozyme diversity in mammals is greatly reduced when we control for phylogenetic dependence, which suggests that phylogenetic effects may be important in this dataset. However, Mulligan *et al.* [8] have previously shown that there is a correlation between mtDNA and allozyme diversity across mammalian orders (see also [9]). Since, most mammalian orders are related to each other by a star-phylogeny, they should be largely independent from one another. There are two explanations for why our results differ from Mulligan *et al.* [8]. First, averaging allozyme and mtDNA diversities across species within orders, as Mulligan et al. have done, is likely to reduce the variance, which is likely to increase the power of the analysis. Second, it may be that most of the variation in effective population size is between orders, not species within orders. It seems likely that on balance there is a correlation between allozyme and mtDNA diversity in mammals which is independent of phylogeny.

Although, the relationships between mitochondrial and allozyme diversities could be a consequence of a correlation between the effective population sizes of the mitochondrial and nuclear genomes, it could also be due to variation in the mutation rate; for example, a change in generation time could change the rate of mutation per generation in both the nuclear and mitochondrial genomes, as we see in plants [10]. Therefore to further investigate whether there is variation in the effective population of the mitochondrial genome we tested whether the apparent effectiveness of natural selection on non-synonymous mutations was correlated to the level of mitochondrial diversity. We did this by testing whether there is a correlation between ψ = *P_n_*/(*P_s_*+1) and θ_s_ in a manner which controls for the obvious non-independence of the two variables. We remove the non-independence by splitting *P_s_* in to two independent parts, but as a consequence of this, all correlations have to be performed twice, once for ψ_1_ versus θ_s2_ and once for ψ_2_ versus θ_s1_. We only present the correlations of ψ_2_ versus θ_s1_ since the complementary correlations are very similar.

**Figure 2:**
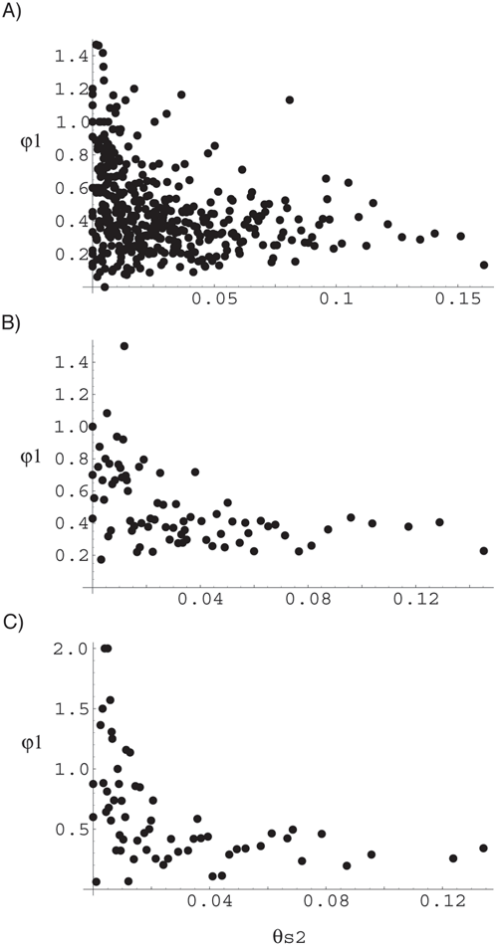
The correlation between ψ_2_ and θ_s1_ when species are aggregated into groups of 4 across (a) all species, (b) within mammals, and (c) within fish.

Overall we observe a non-significant negative correlation between ψ and θ_s_ (table 2), but within mammals and fish the correlation is strong and highly significant, even if we correct for multiple tests using a Bonferroni correction. If we aggregate species into groups of four to reduce the variance in ψ, we find that there is a negative correlation between ψ and θ_s_ in all groups, with the correlation being significant in many of them, with fish, mammals and spiders being significant after correction for multiple tests (Table 2; Figure 2). However, even if we remove these three groups there is still evidence of a significant correlation between ψ_1_ and θ_s2_ for the remaining groups if we combine probabilities (p<0.0001); we can even remove Echinoderms and Molluscs, which are marginally significant individually, and the combined probability value is still significant for the remaining groups (p = 0.003). Qualitatively similar results are obtained for other group sizes (Table S1).

**Table 2:** The correlation between ψ and θ_s_ for mitochondrial DNA.

Dataset	ψ_1_ v θ_s2_			ψ_1_ v θ_s2_ – groups of 4			ψ_1_ v θ_s2_ – phylogenetically independent		
	n	r_s_	p-value	n	r_s_	p-value	n	r_s_	p-value
All	1711	−0.035	0.15	428	−0.326	<0.0001	374	−0.11	0.042
Amphibians	91	0.038	0.72	23	−0.173	0.43	18	0.29	0.24
Birds	217	0.080	0.24	55	−0.190	0.17	56	−0.03	0.84
Chelicerata	23	−0.44	0.035	6	−0.943	0.0048	6	−0.60	0.24
Crustacea	63	0.010	0.94	16	−0.362	0.17	10	−0.31	0.38
Echinoderms	45	−0.28	0.063	12	−0.525	0.080	12	−0.21	0.50
Fish	241	−0.23	0.0003	61	−0.548	<0.0001	50	−0.30	0.030
Insects	461	0.058	0.22	116	−0.124	0.19	97	−0.15	0.13
Mammals	304	−0.16	0.0058	76	−0.491	<0.0001	67	−0.28	0.020
Mollusca	118	−0.094	0.31	30	−0.423	0.020	22	−0.18	0.43
Reptiles	146	−0.0018	0.98	37	−0.201	0.23	36	0.10	0.54

However, these correlations between ψ and θ_s_ might be due to phylogenetic non-independence. To address this, we reduced the data to phylogenetically independent pairs of species by selecting two species from each genus, for which we had two or more species, and testing for a correlation between the difference in ψ_1_ and the difference in θ_s2_. Overall there is a significant correlation between the difference in ψ_1_ and the difference in θ_s2_ (table 2), and the correlation is positive in 8 out of the 10 groups (p = 0.055). Individually mammals and fish show marginally significant correlations, although neither of these significant results survive correction for multiple tests. However, if we combine probabilities across all groups we find that the correlation is highly significant (p = 0.006). It therefore seems that there is a correlation between the effectiveness of selection on non-synonymous mutations and effective population size, even if we control for phylogenetic non-independence.

## Discussion

We have shown that there is a positive correlation between synonymous diversity in mtDNA and allozyme diversity across animal species, even when phylogenetic non-independence is controlled for. This correlation is present in all groups of animals we have considered, and is significant in several of them. We have also shown that the apparent effectiveness of selection on non-synonymous mutations in the mitochondrial genome is correlated to levels of mitochondrial diversity. Both of these observations are highly consistent with variation in the effective population size of mtDNA. However, the correlation between allozyme and mitochondrial diversities could be a consequence of correlated mutation rates in the nuclear and mitochondrial genomes. It is less easy to explain the correlation between ψ and θ_s_ without invoking variation in the effective population size; one would need ψ, and hence the distribution of fitness effects, to be correlated to the mutation rate per generation, and there seems no obvious reason why these two variables should be correlated, except through variation in the effective population size. It therefore seems that there is variation in the effective population size of mtDNA in many, if not all, groups of animals that we have considered.

However, although we have provided evidence for variation in the effective population size, it is still notable that levels of diversity, whether nuclear or mitochondrial, differ remarkably little between species. For example the average mitochondrial synonymous diversity of primates and rodents differs by less than 4-fold and yet their census population sizes must differ by orders of magnitude. There are several explanations for why diversity might not reflect census population size.

First, as Maynard Smith and Haigh [11] first suggested, neutral diversity might be held in check by the effects of adaptive substitutions, which purge diversity as they sweep through the population. If the rate of adaptive evolution is limited by the supply of mutations then the level of neutral diversity is a product of two conflicting processes; as the population size increases so neutral diversity tends to increase, but at the same time the number of adaptive substitutions increases and this decreases diversity. Gillespie [5] has shown that these processes tend to cancel each other out to yield a constant level of neutral diversity across species with very different census population sizes, when there is no recombination. This process is also likely to operate in recombining genomes if the rate of adaptive substitution is fairly high. Bazin et al. [6] show, in support of this “genetic draft” hypothesis, that the neutrality index is significantly lower in invertebrates, which are likely to have higher census population sizes, than vertebrates. However, the neutrality index largely depends upon two factors, the proportion of substitutions that are adaptive, which reduces the neutrality index, and the proportion of polymorphisms that are slightly deleterious, which increases the index [12]. It is therefore possible that invertebrates have lower neutrality indices because they have a smaller proportion of slightly deleterious mutations, not because the rate of adaptive evolution is higher. Furthermore, the degree to which a genome is affected by genetic hitch-hiking depends on the *number* of adaptive substitutions per generation, not the *proportion* of substitutions that are adaptive. Therefore the NI may not be strongly correlated to the rate of genetic draft. It has also been suggested that the difference between the NI values of vertebrates and invertebrates could be due to compositional differences and the difficulties of correcting for multiple substitutions [13].

Second, background selection could potentially cause the level of diversity to be independent of the population size [6]; as the effective population size increases, so selection becomes more effective, increasing the number of deleterious mutations that are removed from the population, and hence reducing the effective population size. However, Bazin et al. [6] have shown that this model does not predict that the effective population size will be independent of census population size under realistic parameter values.

The background selection model depends upon the indirect effect of selection on neutral diversity. However, if the sites being considered are subject to selection then the proportion of mutations that are effectively neutral will depend directly on the effective population size. As the population size increases so the level of neutral diversity increases, but at the same time the proportion of mutations that are effectively neutral decreases. Under certain conditions the increase in diversity, due to an increase in population size, can be exactly offset by an increase in the effectiveness of selection, to yield a constant level of DNA diversity [14]. However, this model only works when some of the mutations are slightly deleterious, and there is currently little evidence of selection on synonymous codon use in mitochondrial DNA in any organism [15].

Finally, the mutation rate per generation and census population size might be negatively correlated. This is not unlikely, since species with short generation times might be expected to have large population sizes and low mutation rates *per generation*, even if they might have high mutation rates *per year*. For example, the nuclear mutation rate *per year* is ∼5-fold higher in rodents than in hominids, but the mutation rate *per generation* is ∼10-fold lower [16]. To investigate this further we estimated the mutation rate per year and per generation in mitochondrial DNA from the level of synonymous divergence at 4-fold degenerate sites, *d_4_*, for whole mitochondrial genome sequences from several pairs of animals for which we have well estimated divergence times, and a rough estimate of generation time. We assume here that synonymous mutation are neutral and that the synonymous divergence gives an estimate of the mutation rate; although, there is a discrepancy between mutation rate estimated from pedigrees and the level of synonymous divergence, this is probably a consequence of the methods used to infer the pedigree mutation rate [17]. There is evidence that the mutation rate varies between sites within the protein coding complement of the mitochondrial genome [18], but the degree to which the mutation rate varies is as yet unknown. We therefore estimated the divergence assuming that sites evolved at the same rate and under a gamma distribution of rates.

As Nabholz *et al.* [19] have recently shown, there is variation in the mutation rate per year between mammalian species in mtDNA; we estimate that under the equal rates model, mutation rates per year vary by just under 4-fold (table 3); if we allow some variation in the mutation rate between sites then the variation between species increases, but not greatly, unless the variation between sites is very large. For example, with an exponential distribution of rates (a gamma distribution with a shape parameter of one), the variation in the mutation per year is about 4.5 fold between pairs of species, and with a shape parameter of 0.5 it increases to 11-fold; it should be noted that with a gamma shape parameter of 0.5, the top 5% of sites mutate ∼1000× faster than the bottom 5% of sites, so this represents extreme variation in the mutation rate.

**Table 3:** The estimated rate of mutation per generation in mitochondrial DNA in selected mammals.

			Equal rates model			Gamma rates model (shape = 1.0)		
Species	Divergence time (MYR)	Generation time (Yrs)	d_4_	mutation per year (×10^−6^)	mutation rate per generation (x 10^−6^)	d_4_	mutation per year (×10^−6^)	mutation rate per generation (x 10^−6^)
Human-chimpanzee	8.3	25	0.343	0.0207	0.517	0.581	0.0350	0.875
Macaque-capuchin	40	11	1.13	0.0141	0.155	3.89	0.0486	0.535
Sheep-cow	24	6	0.759	0.0158	0.0948	2.32	0.0483	0.290
Dog-cat	53	4	1.24	0.0117	0.0469	4.01	0.0378	0.151
Mouse-rat	12	0.5	1.10	0.0460	0.0230	3.49	0.145	0.0727

**Figure 3:**
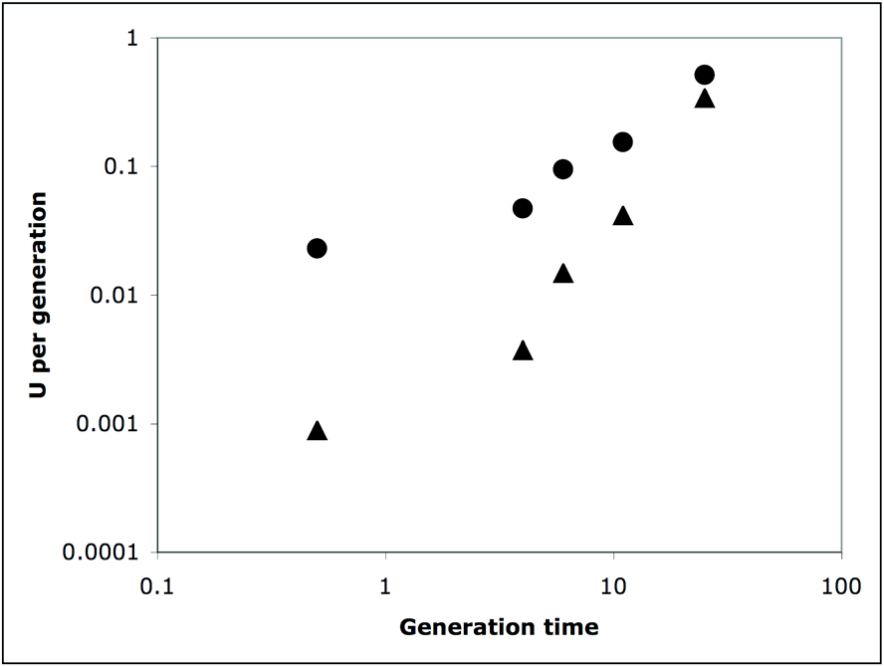
The estimated mutation rate per generation in mtDNA versus generation time. The triangles are for the equal rates model the circles for the gamma rates model assuming a shape parameter of 1.0.

Despite the fact that there is generally a negative correlation between generation time and the mutation rate *per year*, there is a positive correlation between generation time and the mutation rate *per generation* (table 3, figure 3). This is true even if we allow the gamma shape parameter to be 0.5 (results not shown). Hence, since generation time is negatively correlated to population size [20] we might expect population size and the mutation *per generation* to be negatively correlated and for the two factors to cancel each other out, yielding a fairly constant level of diversity.

Since the mutation rate per generation is positively correlated to generation time, and generation time is negatively correlated to population size [20], the apparent constancy of nuclear and mitochondrial diversity across species may hide large variation in effective population size. To investigate this we used our estimates of the mutation rate per generation to derive rough estimates of the average effective population size of mitochondrial DNA. Since many of the species for which we have estimated the mutation rate are domesticated we take the average diversity for the family or sub-family containing the species for which we have estimated the mutation rate. The level of mitochondrial DNA diversity differs very little across these families, which means, given that the mutation rate per generation does vary considerably, that effective population sizes vary by at least an order of magnitude (table 4). Surprisingly we estimate that carnivores have quite large effective population sizes; this may be because our sample in this analysis has a large number of small carnivores, whereas the analysis of allozyme and mtDNA diversities is more biased towards large carnivores. It should be emphasised that the estimates of effective population size are approximate; but give us a guide to the likely variation in the effective population size of mitochondrial DNA that there is in mammals. It therefore seems that there is considerable variation in effective population size, in mammals at least, and that the apparent constancy of diversity across mammalian species is due to a negative correlation between the mutation rate per generation and the effective population size. Whether this is case in other groups of animals remains to be ascertained.

**Table 4:** Estimated mtDNA effective population sizes for selected groups of mammals.

			Equal rates model		Gamma model (shape = 1.0)	
Families, sub-families, genera	number of species	average θ_s_	mutation rate per generation (×10^−6^)	Average N_e_ (female)	mutation rate per generation (×10^−6^)	Average N_e_ (female)
Homo & Pan	2	0.0103	0.517	10,000	0.875	5,900
Catarrhini & Platyrrhini	24	0.0454	0.155	150,000	0.535	42,000
Bovinae & Caprinae	8	0.0504	0.0948	270,000	0.290	87,000
Felidae & Canidae	26	0.0399	0.0469	430,000	0.151	130,000
Murinae	10	0.0335	0.0230	730,000	0.727	230,000

## Materials and Methods

### DNA sequence data

Alignments of mtDNA sequences were kindly provided to us by Eric Bazin. These are an updated compilation previously used by Bazin *et al.* [6]. These datasets were automatically retrieved from Genbank through the Polymorphix [21] database system which looks for homologous sequences from a single species. Sequences are retained by the Polymorphix system if at least two other sequences came from the same study. The sequences were automatically aligned with clustalW [22]. We checked the data and removed all sequences containing premature stop codons. These stop codons may be sequencing errors, null alleles or errors in the alignment. This gave us a dataset of 1712 species (243 fish, 91amphibians, 217 birds, 23 chelicerates, 63 crustaceans, 45 echinoderms, 462 insects, 146 reptiles, 304 mammals and 118 molluscs). For each dataset we computed the number of non-synonymous and synonymous polymorphisms.

### Analysis 1: correlation between mtDNA and allozyme diversity?

In our first analysis we tested whether diversity at synonymous sites in mtDNA was correlated to allozyme diversity. Allozyme heterozygosities were taken from a review by Nevo *et al.* [3]. The level of synonymous diversity per site in mtDNA was estimated using Watterson's estimator
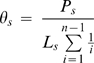
(1)where *P_s_* is the number of synonymous polymorphisms, *L_s_* is the number of synonymous sites and *n* is the number of sequence sampled. It makes sense in this context to calculate θ_s_ per physical site [23] so we took the number of synonymous sites as 30% of the total length of the sequence.

To control for phylogenetic non-independence we constructed the phylogenies for the species for which we had both mtDNA and allozyme data, using a combination of traditional systematics, published molecular phylogenies and expert advice (Text S1, Figures S1, S2, S3, S4, S5, S6). For each group of animals we paired species to form a set of independent contrasts and considered the correlation between the difference in allozyme heterozygosity and mitochondrial diversity.

### Analysis 2 : variation in the efficiency of selection

In our second analysis we tested whether the strength of purifying selection on non-synonymous mutations was correlated to the effective population size of the mtDNA across species. We can do this by considering the correlation between *P_n_/P_s_* and θ_s_, where *P_n_* and *P_s_* are the numbers of non-synonymous and synonymous mutations respectively, and θ_s_ is Watterson's estimator of the synonymous diversity. *P_n_/P_s_* is a measure of the strength of selection acting on deleterious non-synonymous mutations; when *P_n_/P_s_* is large selection is relatively weak. Since, θ_s_ equals 2*N_e_u* for neutral mutations, θ_s_ is a measure of the effective population size that the polymorphism data has experienced; i.e. *P_n_/P_s_* and θ_s_ are measured over the same time-scale.

However, there are three problems. First *P_s_* and θ_s_ are not independent since θ_s_ depends on *P_s_*; in fact we would expect *P_n_/P_s_* and θ_s_ to be negatively correlated just through sampling error. To overcome this problem we split *P_s_* into two independent values by generating a random binomial variate with sample size *P_s_* and probability value of 0.5 (this is akin to dividing the sequence into odd and even codons):
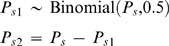
(2)


By using separate *P_s_* values to estimate ψ and θ_s_ we remove the non-independence between these variables.

The second problem is that *P_n_/P_s_* is undefined if *P_s_* = 0, and the third problem is that *P_n_/P_s_* can be an overestimate of the expected value of *P_n_/P_s_*: i.e. E(*Pn/Ps*)>E(*P_n_*)/E(*P_s_*) for moderate values of E(*P_s_*). Both of these problems can be overcome by considering the correlation between ψ and θ_s_ where
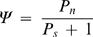
(3)


Assuming that *P_s_* is Poisson distributed It can be shown that ψ underestimates E(*P_n_*)/E(*P_s_*) when E(*P_s_*) is less than three but is essentially unbiased (less than 5% below the value of E(*P_n_*)/E(*P_s_*)) for higher values of E(*P_s_*)). We denote ψ and θ_s_ calculated using *P_sx_* as ψ_x_ and θ_sx_. We ran simulations to test whether the method was unbiased. We simulated the extreme case of no recombination by generating genealogies under a standard neutral model then distributing non-synonymous and synonymous polymorphisms on the genealogy. We then split the number of synonymous polymorphisms as above and calculated ψ_1_ and θ_s2_. We found, as expected, that when E(*P_s_*) was small, the procedure tended to generate a small positive correlation between ψ_1_ and θ_s2_; this is because ψ is underestimated when E(*P_s_*) is small. As E(*P_s_*) increased so this positive correlation disappeared to leave no correlation between ψ_1_ and θ_s2_. The method is therefore slightly conservative in that it tends to produce a positive correlation.

Unfortunately, our estimate of ψ is subject to considerable variance because many of the datasets contain relatively little polymorphism. We therefore summed data across species in the following manner. First, we ranked species according to the value of θ_s2_; we then grouped species according to this ranking into groups of size *n*. For each group we averaged the values of θ_s2_ and summed the values of *P_n_* and *P_s1_* before calculating ψ_1_. We then considered the correlation between ψ_1_ and θ_s2_ across groups; as expected similar results were obtained using ψ_2_ and θ_s1_. We performed this analysis for groups of size 2, 4, 8 and 16. Results were qualitatively similar across all group sizes (Table S1), we therefore present the results for groups of size 4.

Controlling for phylogenetic non-independence in such a large dataset is difficult because knowing the complete phylogeny is problematic. As a consequence we chose one pair of species from each genus to form phylogenetically independent contrasts. Species pairs were chosen according to the length of the sequence in the alignment. If datasets differed by less than 10% in length we took the species with the largest number of individuals sequenced. For each species pair we calculated the difference in ψ_2_ and the difference in θ_s1_, and considered the correlation between these differences. It was not possible to control for phylogeny when we grouped species.

All correlations were performed using Spearman's rank correlation and we combined probabilities using the unweighted Z-method [24].

### Mutation rates and effective population sizes

As part of our analysis we also estimated the mutation rate per generation for mitochondrial DNA by considering the level of synonymous divergence between selected pairs of animal species for which we have an estimate of the divergence and generation times; i.e. we assume that synonymous mutations are neutral. These pairs of species are human-chimpanzee, macaque-capuchin, cow-sheep, dog-cat and mouse-rat. We took divergence dates from a recent review of the fossil evidence [25], taking the average of the maximum and minimum dates. Where fossil dates were not available we used divergence dates inferred from locally calibrated molecular clocks as compiled by Keightley and Eyre-Walker [16]. Estimates of generation times were taken from Keightley and Eyre-Walker [16].

We downloaded the complete mtDNA sequences for each of these species and extracted the protein coding sequences; overlapping regions were removed. Restricting ourselves to codons in which the amino acid is the same in both species we estimated the divergence at 4-fold degenerate synonymous sites, *d_4_*, using the method of Tamura and Nei [26] which takes into account base composition bias and allows the rates of C<->T and A<->G transitions to differ, as well as the rate of transversion. Multiplying *d_4_* by the generation time and dividing it by twice the divergence time gives us an estimate of the mutation rate per generation.

### Effective population size

Since the θ_s_ is expected to be equal to 2*N_e_u* for neutral mutations in mtDNA, where *u* is the mutation rate per generation and *N_e_* is the effective population size of females, we can estimate the effective population size of mitochondrial DNA given our estimate of the mutation rate per generation. We do not have diversity data for many of the species for which we have estimated the mutation rate per generation; furthermore many of these species are domesticated animals so their diversity is unlikely to reflect that of their wild relatives. We therefore took the average mtDNA diversity across the following groups: for human-chimp we averaged across the genera *Homo* and *Pan*; for macaque-capuchin we averaged across the species within *Platyrhini* and *Catorrhini*, excluding *Pan* and *Homo*; for cow-sheep we averaged across the species within *Bovinae* and *Caprinae*, for dog-cat across the species within *Canidae* and *Felidae* and for mouse-rat within the *Murinae*.

## Supporting Information

Table S1 The correlation between ψ2 and θs1 for mitochondrial DNA where species are ranked according to their θs1 and then grouped into groups of size n; the last group, of genes with the highest θs1 may contain fewer species if the number of species is not perfectly divisable by the group size.
(0.06 MB DOC)

Figure S1 Phylogeny of amphibians.
(0.04 MB PDF)

Figure S2 Phylogeny of birds.
(0.04 MB PDF)

Figure S3 Phylogeny of fish.
(0.05 MB PDF)

Figure S4 Phylogeny of insects.
(0.04 MB PDF)

Figure S5 Phylogeny of mammals.
(0.05 MB PDF)

Figure S6 Phylogeny of reptiles.
(0.04 MB PDF)

Text S1 Supporting Information
(0.07 MB DOC)
