# Supplementary material for: Evidence for Variation in the Effective Population Size of Animal Mitochondrial DNA
Source: PLoS One. 2009 Feb 9;4(2):e4396. doi: 10.1371/journal.pone.0004396 (PMC2635931; doi:10.1371/journal.pone.0004396)
Supplement: Table S1 — The correlation between ψ2 and θs1 for mitochondrial DNA where species are ranked according to their θs1 and then grouped into groups of size n; the last group, of genes with the highest θs1 may contain fewer species if the number of species is not perfectly divisable by the group size. (0.06 MB DOC) [file pone.0004396.s001.doc]

|  | Group size = 2 | | Group size = 4 | | Group size = 8 | | Group size = 16 | |
| --- | --- | --- | --- | --- | --- | --- | --- | --- |
| Group | n | r_s_ | n | r_s_ | n | r_s_ | n | r_s_ |
|  |  |  |  |  |  |  |  |  |
| Amphibians | 46 | -0.14 | 23 | -0.17 | 12 | -0.32 | 6 | -0.6 |
| Birds | 109 | 0.0054 | 55 | -0.19 | 28 | -0.40 | 14 | -0.38 |
| Chelicerata | 12 | -0.62 | 6 | -0.94 | 3 | -1.0 | 2 | -1.0 |
| Crustacea | 32 | -0.23 | 16 | -0.36 | 8 | -0.29 | 4 | -0.4 |
| Echinoderms | 23 | -0.32 | 12 | -0.52 | 6 | -0.66 | 3 | -1.0 |
| Fish | 121 | -0.43 | 61 | -0.55 | 31 | -0.82 | 16 | -0.85 |
| Insects | 231 | -0.065 | 116 | -0.12 | 58 | -0.091 | 29 | -0.28 |
| Mammals | 158 | -0.38 | 76 | -0.49 | 38 | -0.63 | 19 | -0.81 |
| Molluscs | 59 | -0.22 | 30 | -0.42 | 15 | -0.52 | 8 | -0.60 |
| Reptiles | 73 | -0.16 | 37 | -0.20 | 19 | -0.12 | 10 | -0.2 |

**Supplementary table 1.** The correlation between ψ_2_ and θ_s1_ for mitochondrial DNA where species are ranked according to their θ_s1_ and then grouped into groups of size n; the last group, of genes with the highest θ_s1_ may contain fewer species if the number of species is not perfectly divisable by the group size.
