# Supplementary material for: Evidence for Variation in the Effective Population Size of Animal Mitochondrial DNA
Source: PLoS One. 2009 Feb 9;4(2):e4396. doi: 10.1371/journal.pone.0004396 (PMC2635931; doi:10.1371/journal.pone.0004396)
Supplement: Figure S1 — Phylogeny of amphibians. (0.04 MB PDF) [file pone.0004396.s002.pdf]

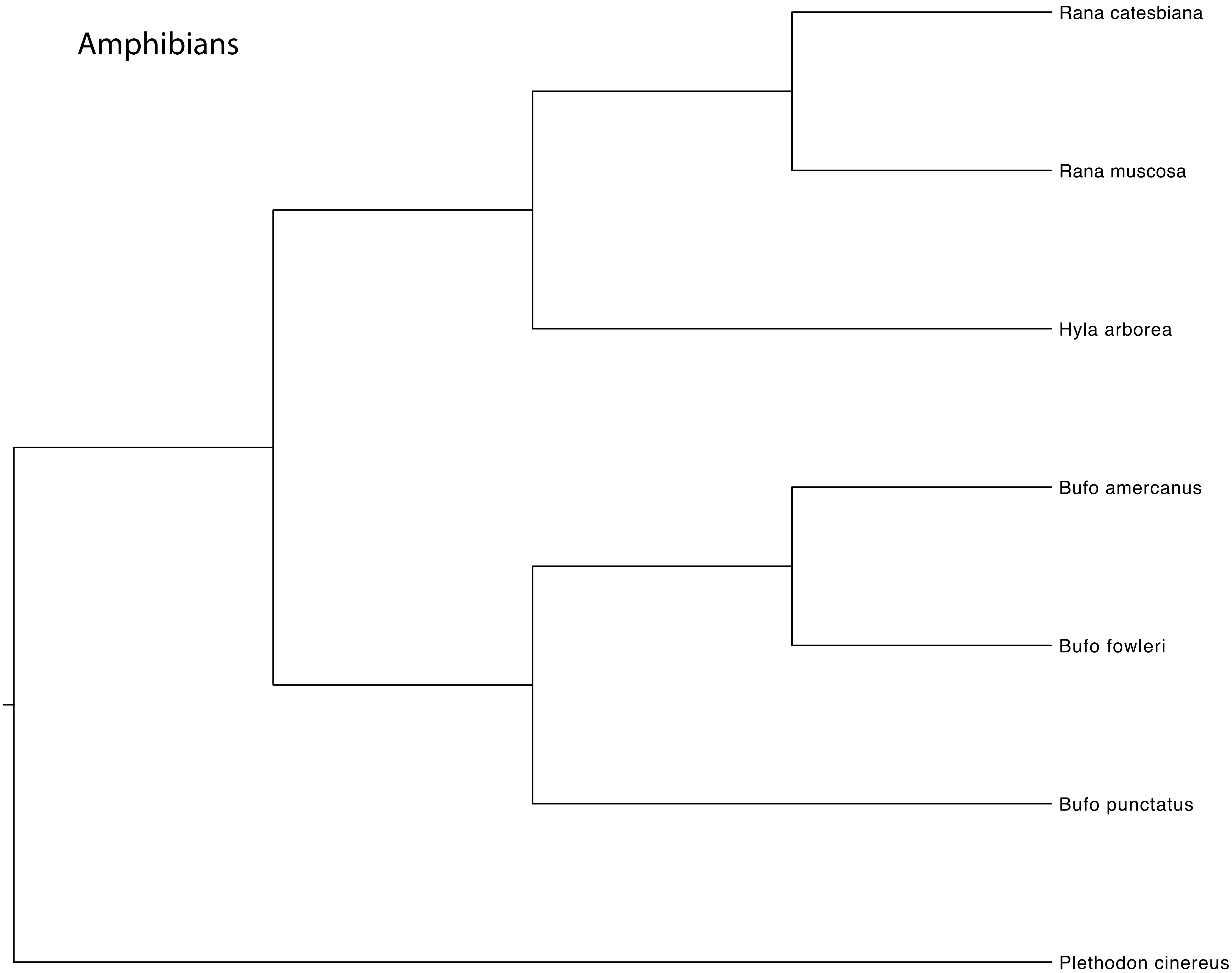
Amphibians
Rana catesbiana
Rana muscosa
Hyla arborea
Bufo amercanus
Bufo fowleri
Bufo punctatus
Plethodon cinereus
