# Supplementary figures and images for: Evidence for Variation in the Effective Population Size of Animal Mitochondrial DNA
Source: PLoS One. 2009 Feb 9;4(2):e4396. doi: 10.1371/journal.pone.0004396 (PMC2635931; doi:10.1371/journal.pone.0004396)

# Birds

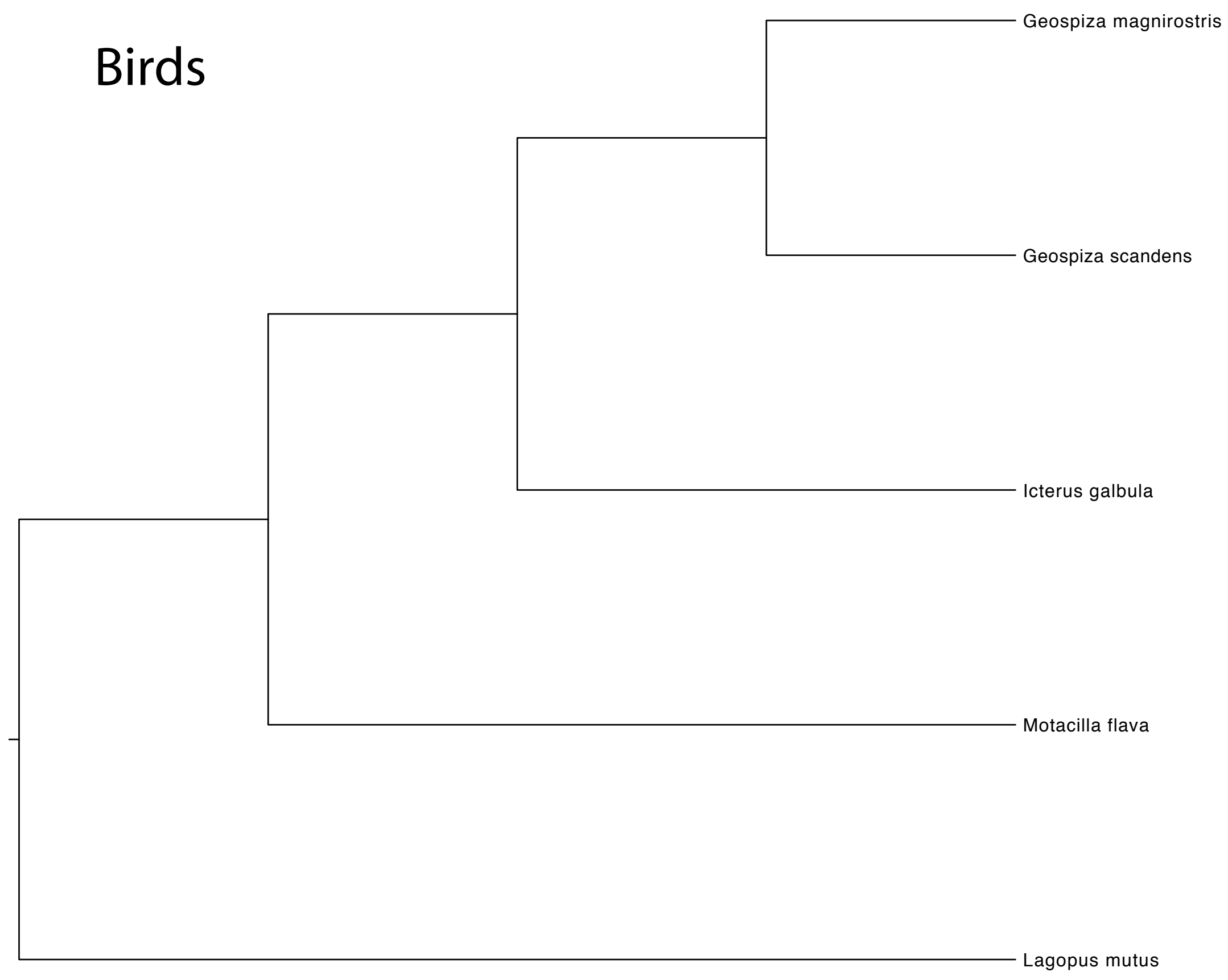

Supplement: Figure S2 — Phylogeny of birds. (0.04 MB PDF) [file pone.0004396.s003.pdf]

# Fish

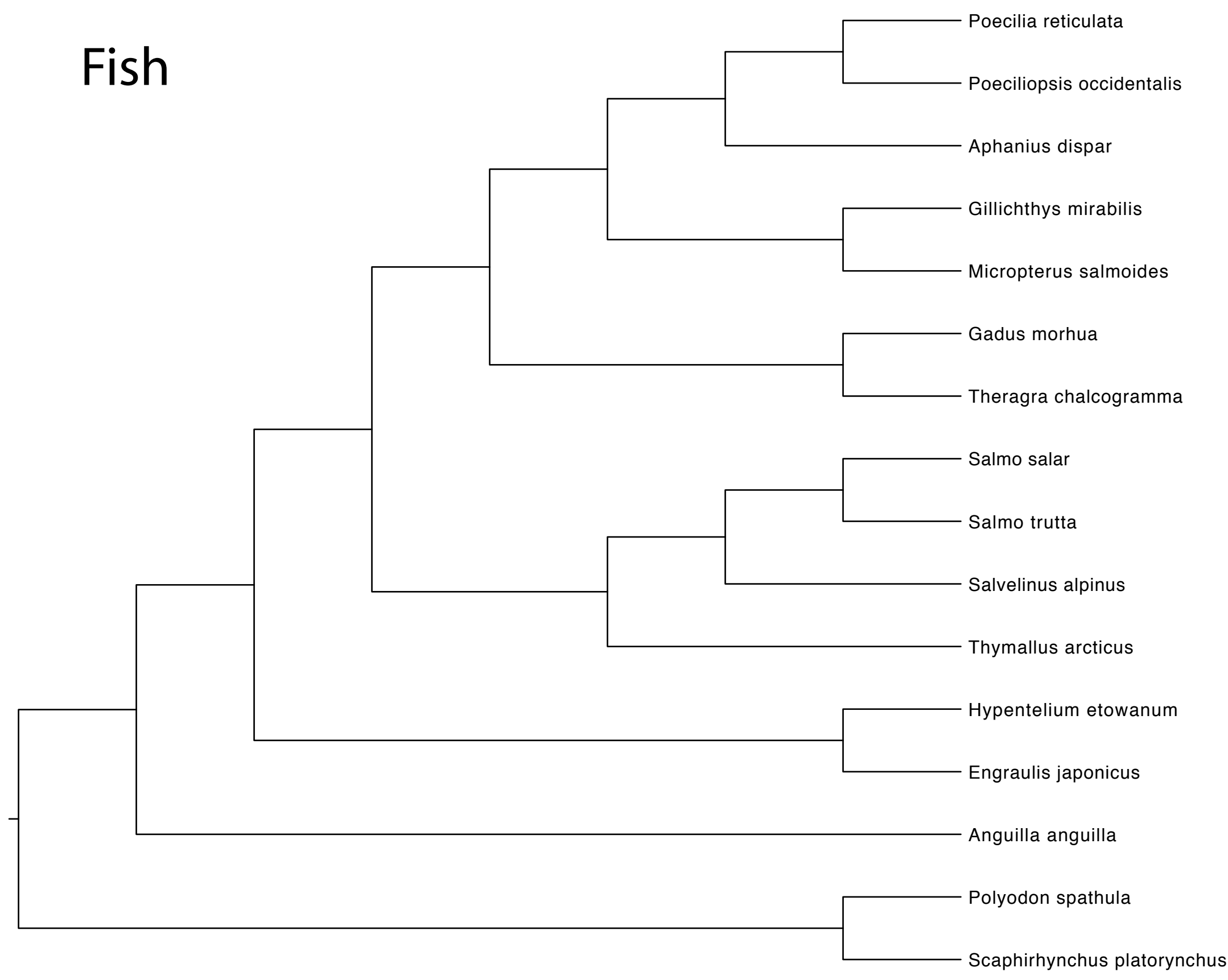

Supplement: Figure S3 — Phylogeny of fish. (0.05 MB PDF) [file pone.0004396.s004.pdf]

# Insects

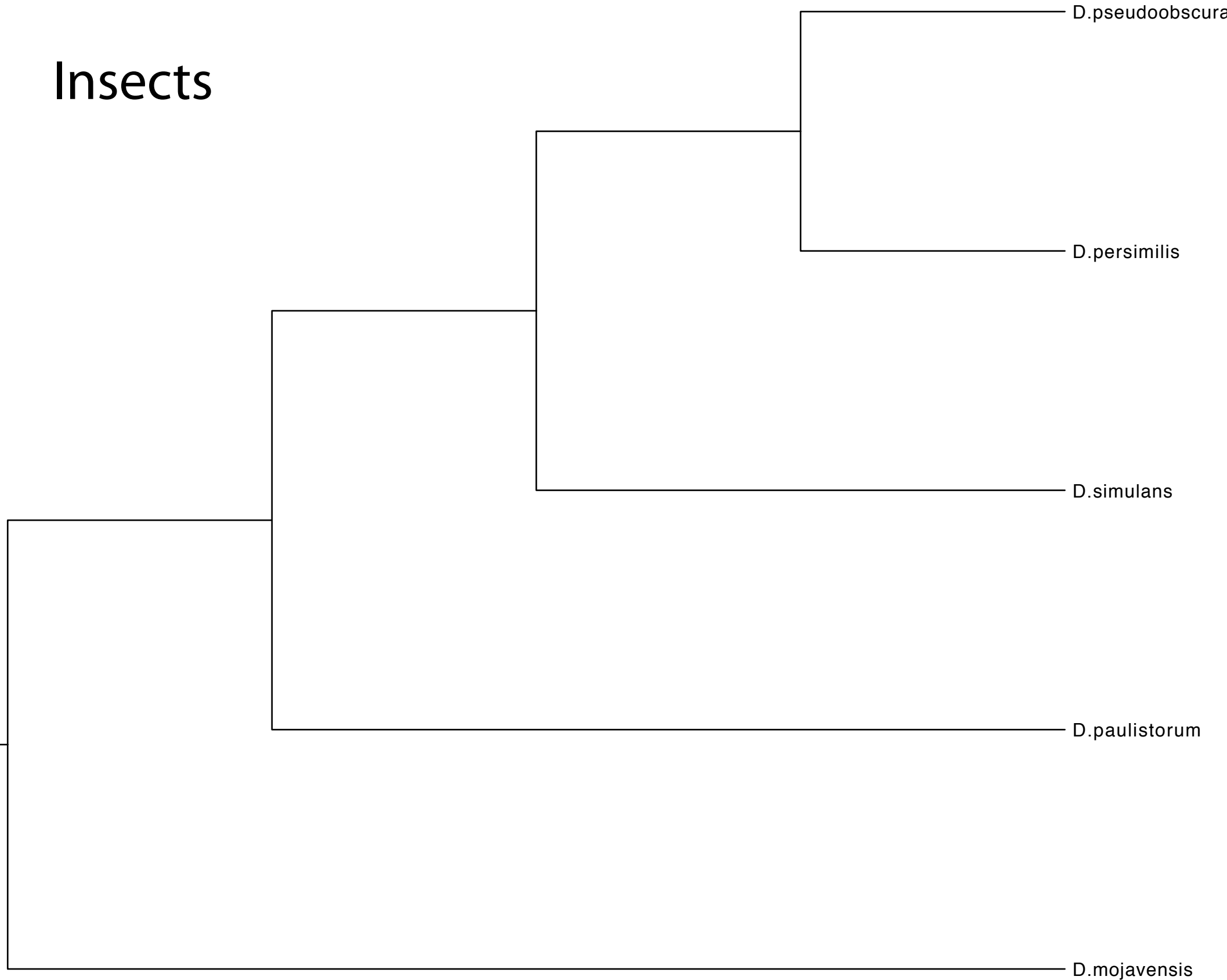

Supplement: Figure S4 — Phylogeny of insects. (0.04 MB PDF) [file pone.0004396.s005.pdf]

# Mammals

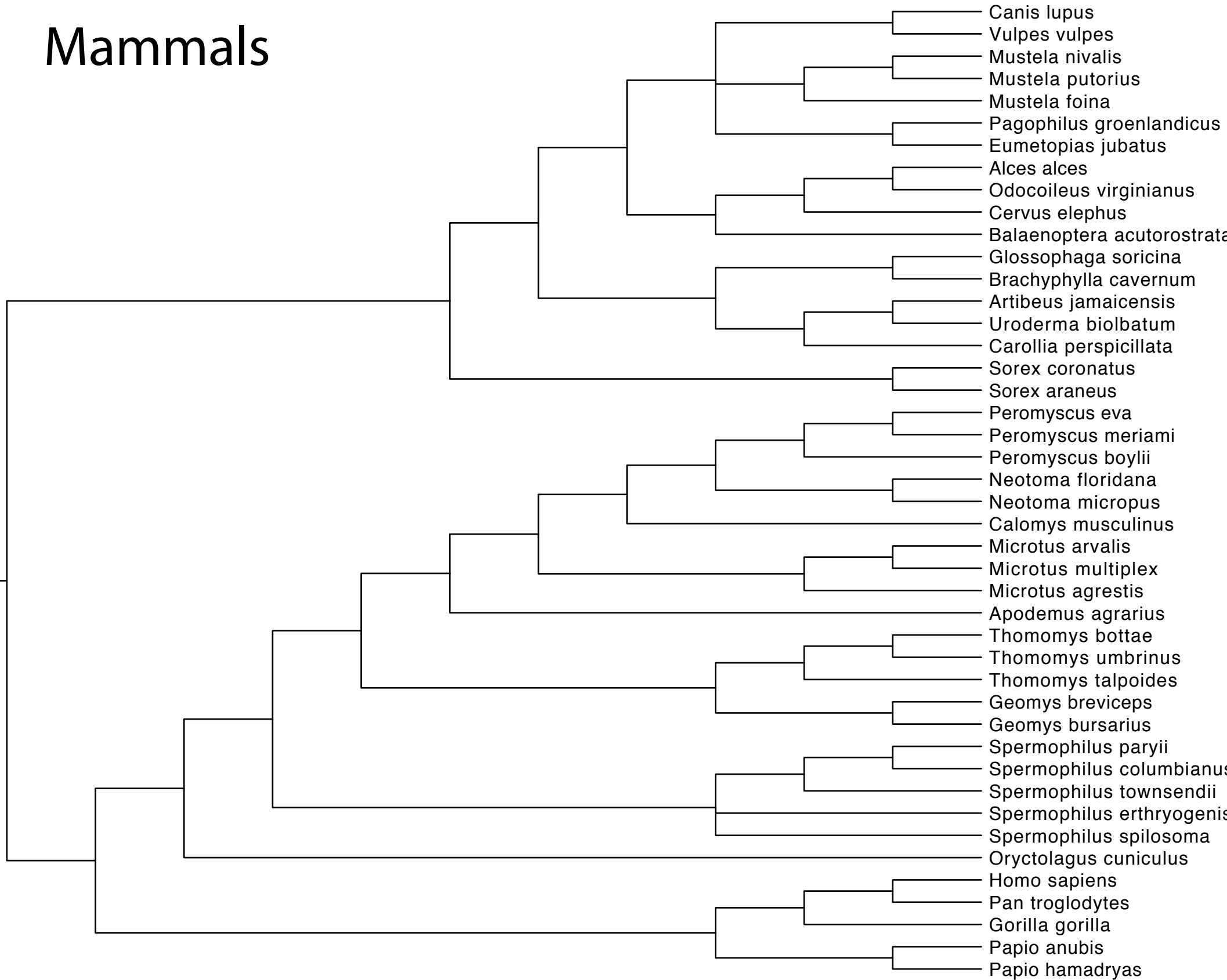

Supplement: Figure S5 — Phylogeny of mammals. (0.05 MB PDF) [file pone.0004396.s006.pdf]

# Reptiles

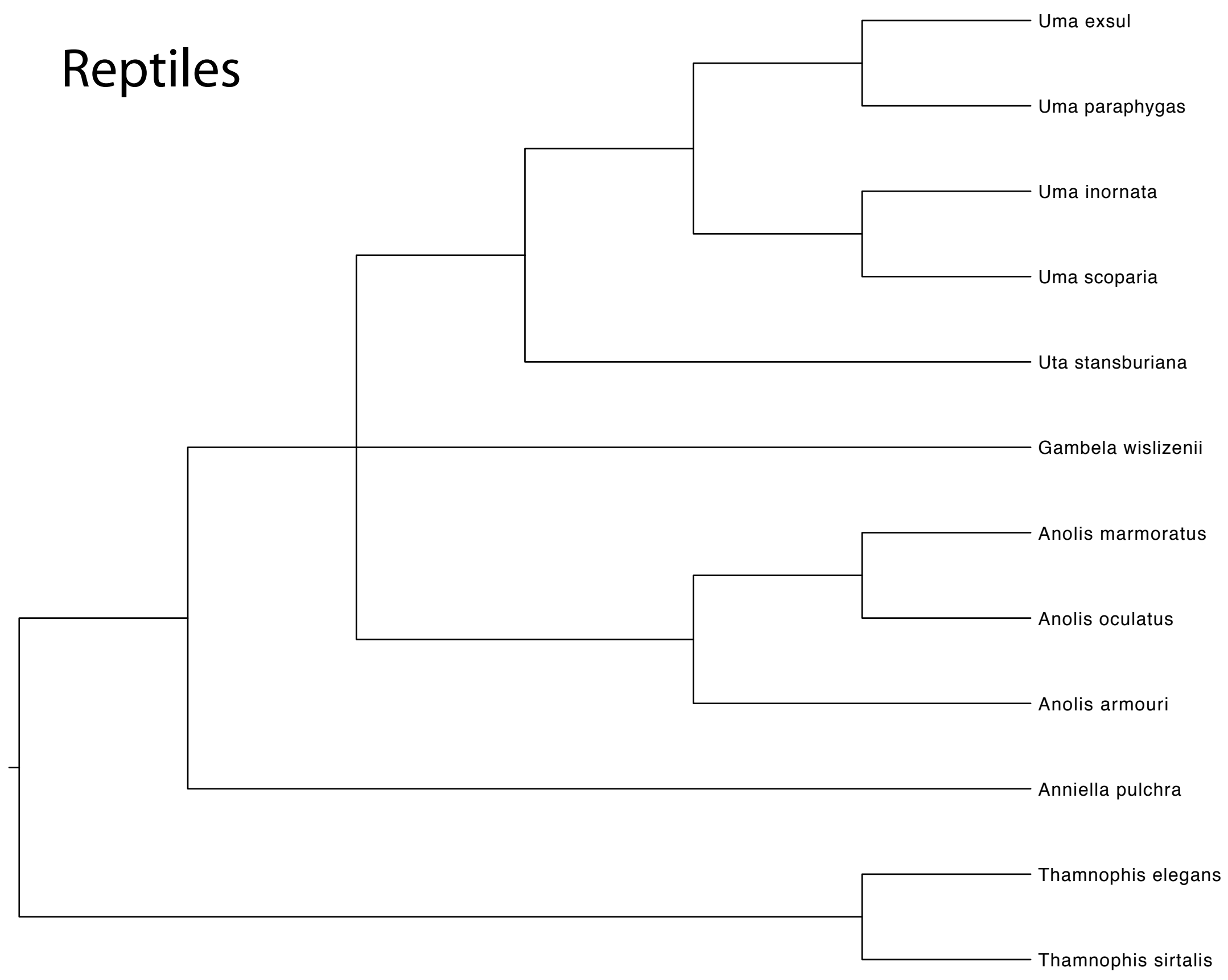

Supplement: Figure S6 — Phylogeny of reptiles. (0.04 MB PDF) [file pone.0004396.s007.pdf]
