# Supplementary material for: Evidence for Variation in the Effective Population Size of Animal Mitochondrial DNA
Source: PLoS One. 2009 Feb 9;4(2):e4396. doi: 10.1371/journal.pone.0004396 (PMC2635931; doi:10.1371/journal.pone.0004396)
Supplement: Text S1 — Supporting Information (0.07 MB DOC) [file pone.0004396.s008.doc]

**Evidence for Variation in the Effective Population Size of Mitochondrial DNA**

Gwenael Piganeau & Adam Eyre-Walker

**Supplementary Information**

In this section we provide a table showing the correlation between ψ_1_ and θ_s2_ for group sizes of 2, 4, 8 and 16. We also provide the phylogenies that we used to construct independent contrasts to test whether allozyme and mitochondrial diversities are correlated independent of phylogeny. The phylogenies were constructed using a combination of phylogenetic information from the NCBI taxonomy browser, primary literature and expert advice.

**Amphibians**

The phylogeny was taken from the NCBI taxonomy browser with additional information for the *Bufo* species provided by Prof Trevor Beebee (University of Sussex, UK).

**Birds**

The phylogeny was taken from the NCBI taxonomy browser.

**Fish**

The phylogeny was taken from the NCBI taxonomy browser with additional information for the salmonids taken from [1].

**Insects**

The phylogeny was taken from [2].

**Mammals**

The basic structure of the phylogeny was taken from [3]. The relationships among the bats was taken from [4]. Among the rodents specific information came for the *Spermophilus*  species from [5], for the *Thomomys* species from [6], for the *Microtus* species from [7], for *Neotoma* species from [8], for *Peromyscus* species from [9].

**Reptiles**

The phylogeny was taken from the NCBI taxonomy browser with additional information for the *Uma* species provided by Prof. Bob Murphy (University of Toronto, Canada).

**Literature**

1. Crespi BJ, Fulton MJ (2004) Molecular systematics of Salmonidae: combined nuclear data yields a robust phylogeny. Mol Phyl Evol 31: 658-679.

2. Powell JR, DeSalle R (1995) Drosophila molecular phylogenies and their uses. Evol Biol 28: 87-138.

3. Murphy WJ, Elzirik E, Johnson WE, Zhing YP, Ryder OA, et al. (2001) Molecular phylogenetics and the origins of placental mammals. Nature 409: 614-618.

4. Baker JR, Hoofer SR, Porter CA, Van Den Bussche RA (2003) Diversification among new world leaf-nosed bats: an evolutionary hypothesis and classification inferred from digenomic congruence of DNA sequence. Occasional Papers, Museum of Texas Tech University 230: 1-32.

5. Harrison RG, Bogdanowicz SM, Hoffmann RS, Yensen E, Sherman PW (2003) Phylogeny and evolutionary histroy of the ground squirrels (Rodentia: Marmotinae). J Mam Evol 10: 249-276.

6. Smith MF (1998) Phylogenetic relationships and geographic structure in pocket gophers in the genuc *Thomomys*. Mol Phyl Evol 9: 1-14.

7. Jaarola M, Martinkova N, Gunduz I, Brunhoff C, Zima J, et al. (2004) Molecular phylogeny of the speciose vole genus Microtus (Arvicolinae, Rodentia) inferred from mitochondrial DNA sequences. Mol Phylogenet Evol 33: 647-663.

8. Edwards CW, Fulhorst CF, Bradley RD (2001) Molecular phylogenetics of the Neotoma albigula species group: further evidence of a paraphyletic assemblage. J Mam 82: 267-279.

9. Riddle BR, Hafner DJ, Alexander LF (2000) Phylogeography and sustemtics of the Peromyscus eremicus species group and the historical biogeography of the North American warm regional deserts. Mol Phyl Evol 17: 145-160.

**Figure S1.** The phylogeny of amphibians.

**Figure S2.** The phylogeny of birds.

**Figure S3.** The phylogeny of fish.

**Figure S4.** The phylogeny of insects.

**Figure S5.** The phylogeny of mammals.

**Figure S6.** The phylogeny of reptiles.
